# Genome Sequence of *Vibrio* VPAP30, Isolated from an Episode of Massive Mortality of Reared Larvae of the Scallop *Argopecten purpuratus*

**DOI:** 10.1128/genomeA.00745-15

**Published:** 2015-07-09

**Authors:** Rodrigo Rojas, Claudio D. Miranda, Jaime Romero, Freddy Asenjo, Katherinne Valderrama, Cristopher Segovia, Juan A. Ugalde, Javier Santander

**Affiliations:** a Laboratorio de Biotecnologia, Instituto de Nutricion y Tecnologia de Alimentos, Universidad de Chile, Santiago, Chile; b Departmento de Acuicultura, Laboratorio de Patobiología Acuática, Universidad Católica del Norte, Coquimbo, Chile; c Center for Genomics and Bioinformatics, Faculty of Sciences, Universidad Mayor, Huechuraba, Chile; d Faculty of Sciences, Microbial Pathogenesis and Vaccinology Research Group, Universidad Mayor, Huechuraba, Chile; e Aquaculture Ph.D. Program, Universidad Catolica del Norte, Coquimbo, Chile; f Faculty of Sciences, Integrative Genomics Ph.D. Program, Universidad Mayor, Huechuraba, Chile; g Interdiciplinary Center for Aquaculture Research, Concepcion, Chile

## Abstract

We report here the 5.167-Mbp draft genome sequence of *Vibrio* VPAP30, isolated from an *Argopecten purpuratus* larval culture. *Vibrio* VPAP30 is the etiological agent of a vibriosis outbreak causing a complete collapse of a larval culture of the scallop *A. purpuratus*, which occurred in a commercial hatchery in Chile.

## GENOME ANNOUNCEMENT

The aquaculture of the scallop *Argopecten purpuratus* (Lamarck, 1819) is one of the most important maricultures in Chile (1). Vibriosis, a disease caused by Gram-negative bacterial *Vibrio* species, is one of the mayor health risks for the *A. purpuratus* aquaculture industry (2). *Vibrio* VPAP30 is a virulent strain that causes mass mortalities to the commercially reared larvae *A. purpuratus*, representing a high economic risk for the Chilean pectinid aquaculture industry. The *Vibrio* VPAP30 strain was isolated from settled moribund and dead larvae of *A. purpuratus* during an outbreak that occurred in a commercial hatchery located in the north of Chile. The main clinical signs are bacterial swarms on the margins of the larvae, extension and disruption of the velum, detachment of velum cilial cells, and digestive tissue necrosis of the larvae.

Genomic DNA of *Vibrio* VPAP30 was extracted according to Wilson and Carson (3, 4) and purified using silica (5). The purified DNA was used to prepare a library with the Nextera kit (Illumina, San Diego, CA). High-throughput sequencing of the library was performed using a MiSeq instrument (Illumina) with a 2 × 300-bp paired-end run, using the reagent kit version 3 (600 cycles) at the Center for Genomics and Bioinformatics, Universidad Mayor, Chile. This resulted in 1,465,456 read pairs for a total of 0.87 Gbp. The reads were trimmed using Trimmomatic 0.32 (6). Genome assembly was performed using SPAdes 3.5.0 (7) and resulted in 43 contigs >1 kb (*N*_50_, 416,210 bp; total length, 5,172,363 bp; G+C content, 44.53%). Functional annotation of predicted gene sequences was performed using the NCBI Prokaryotic Genome Annotation Pipeline (2.10 rev 463717) (http://www.ncbi.nlm.nih.gov/genome/annotation_prok) (8). A total of 4,521 coding sequences (CDSs), 83 pseudogenes, 16 rRNAs, and 2 noncoding RNAs (ncRNAs) were predicted by the pipeline.

Sequence analysis of the gene that codifies for the 16 rRNA gene showed a high degree of sequence identity (>99%) with other unclassified *Vibrio* strains isolated from bivalves (identification 440576560, 261526736, 507718893, and 452084911).

### Nucleotide sequence accession numbers. 

This whole-genome shotgun project has been deposited at DDBJ/EMBL/GenBank under the accession no. LBLS00000000. The version described in this article is version LBLS01000000.
